# Automated Synthesis and Visualization of a Chemotherapy Treatment Regimen Network

**Published:** 2013

**Authors:** Jeremy Warner, Peter Yang, Gil Alterovitz

**Affiliations:** a Department of Medicine, Division of Hematology & Oncology, Vanderbilt University, Nashville, TN, USA; b Department of Biomedical Informatics, Vanderbilt University, Nashville, TN, USA; c Department of Medicine, Division of Hematology & Oncology, Beth Israel Deaconess Medical Center, Boston, MA, USA; d Center for Biomedical Informatics, Harvard Medical School, Boston, MA, USA; e Children’s Hospital Informatics Program at Harvard-MIT Division of Health Science, Boston, MA, USA; f Department of Electrical Engineering and Computer Science, Massachusetts Institute of Technology, Cambridge, MA, USA

**Keywords:** Computing methodologies, Numerical Analysis, Computer-Assisted, Data Interpretation, Statistical, Medical Informatics Applications

## Abstract

Cytotoxic treatments for cancer remain highly toxic, expensive, and variably efficacious. Many chemotherapy regimens are never directly compared in randomized clinical trials (RCTs); as a result, the vast majority of guideline recommendations are ultimately derived from human expert opinion. We introduce an automated network meta-analytic approach to this clinical problem, with nodes representing regimens and edges direct comparison via RCT(s). A chemotherapy regimen network is visualized for the primary treatment of chronic myelogenous leukemia (CML). Node and edge color, size, and opacity are all utilized to provide additional information about the quality and strength of the depicted evidence. Historical versions of the network are also created. With this approach, we were able to compactly compare the results of 17 CML regimens involving RCTs of 9700 patients, representing the accumulation of 45 years of evidence. Our results closely parallel the recommendations issued by a professional guidelines organization, the National Comprehensive Cancer Network (NCCN). This approach offers a novel method for interpreting complex clinical data, with potential implications for future objective guideline development.

## Introduction

Conventional systematic review and meta-analysis are aggregating approaches with a goal of making unifying conclusions based upon multiple independent studies [1]. The traditional meta-analytic approach is generally limited by the requirement that the comparator arms and outcome measures are the same, e.g. progression free survival (PFS) on drug A to PFS on drug B [2]. The traditional meta-analytic approach is challenged by complex scenarios, such as the treatment of cancer, where multiple treatment options with disparate measures of outcome have been tested over the years. In parallel with this increase in complexity, the issuance of clinical management guidelines has increased dramatically over the past years and decades. Most guidelines are derived from collaborations of clinical experts and are therefore subject to subjective interpretation of data. Furthermore, guidelines must be constantly updated due to introductions of new evidence; one published estimate of guideline “half-life” is only 5.5 years [3].

Several approaches have been suggested to meet the need of rigorous objective comparison of multiple treatments used in a common context. These approaches are generally referred to as “network meta-analyses”. Network meta-analysis evaluates multiple treatments and determines the relationships among them, offering a powerful objective solution to this complicated medical need, despite considerable methodological challenges [4, 5]. In this paper, we propose a simplified approach to the construction and display of a meta-analytic network for chemotherapy regimens, with a goal of conveying maximum information about the quality of outcome comparisons, the comparative value of particular regimens, and the relevance of older published regimens to contemporary practice.

## Materials and Methods

### Pilot Use Case

To demonstrate our proposed approach, we selected a condition with a relatively limited number of commonly used treatments, chronic myelogenous leukemia (CML). We limited our evaluation to published randomized clinical trials (RCTs) investigating primary (first-line) treatments of newly-diagnosed chronic-phase CML. These were first identified through a curated database of chemotherapy regimens at HemOnc.org (http://hemonc.org), a hematology/oncology wiki actively maintained by physicians. The publications identified were manually searched to identify further regimens; a PubMed query for the Medical Subject Headings (MeSH) “Leukemia, Myelogenous, Chronic, BCR-ABL Positive” and “Randomized Controlled Trial [Publication Type]” was also conducted. The results of this analysis were compared to the recommendations provided by the NCCN Guidelines® [6].

### Graph Attributes

A network graph was subsequently created, with *v* vertices corresponding to substantively identical chemotherapy regimens and *l* edges connecting regimens which were directly compared in the published RCTs. When more than one RCT compared the same regimens, edges were duplicated. Vertices were depicted as circular nodes, and edges as solid lines. The network layout was first automatically determined using the Kamada-Kawai force-based algorithm, with subsequent manual modification to maximize readability [7]. In order to enhance the information value of the graph, the appearance of the nodes and edges was enhanced in a systematic way, as follows:

#### Node Size and Coloration

Nodes were automatically sized proportionally to the total number of patients who received the specified regimen. Nodes were colored using a gradated three-color system, with red connoting an inferior treatment regimen, green a superior treatment regimen, and yellow a treatment regimen of equivocal value. This value, ${\hat{v}}_{n}$ was calculated by holding a series of *m* “contests” with the immediately adjacent vertices, based on the published outcome findings. The three possible outcomes *E* of each contest are:

- Win (*E* = 1): superiority, as defined by an improved outcome with p-value ≤0.05.
- Lose (*E* = −1): inferiority, as defined by an inferior outcome with p-value ≤0.05.
- Tie (*E* = 0): either an outcome with a non-significant p-value *or* an equivalent outcome as defined by formal non-inferiority, with p-value ≤0.05.

*E* was further multiplied by a “relative value measure” *RV*, representing the quality of the measured outcome: 1.0 for a weak surrogate measure (e.g. response rate); 1.25 for a strong surrogate measure (e.g. PFS); 1.5 for overall survival. Finally, the average of the sum of the products of these values was multiplied by the logarithm of the total patients in all contests involving the vertex, as shown in Equation (1):

(1) $${\hat{v}}_{n}=\frac{\Sigma_{y=1}^{m}RV_{y}\times E_{y}}{m}\times \log(N_{G}[v_{n}])$$

Nodes with negative ${\hat{v}}_{n}$ were automatically colored in the red range, gradating towards yellow for ${\hat{v}}_{n}$ about zero, and towards the green range for positive ${\hat{v}}_{n}$.

#### Edge Width and Coloration

Edge width was automatically sized proportionate to the number of patients being compared across the two treatment regimen vertices for the uniquely referent RCT. If more than one RCT compared the same regimens, the width of each duplicate edge was determined independently. Edges were also colored on a three-color scale, without gradation, to reflect the quality of the measured outcome, which was determined manually: red for weak surrogates (e.g. response rate); yellow for strong surrogates (e.g. PFS); green for overall survival.

#### Node and Edge Aging Effects

In order to convey information about how recently a regimen was formally evaluated, transparency was automatically assigned to older nodes and edges, using the alpha opacity channel. Edges were assigned initial alpha of 1.0 and decayed by 0.1/year to a minimum of 0.2, based upon the “survival analysis” by Shojania et al [3]. Nodes were also assigned initial alpha of 1.0 and decayed in a similar fashion; however, nodes were refreshed to an alpha of 1.0 whenever a new RCT was published which involved the node.

Node alpha was also varied with significant perturbations of the network. Specifically, when new evidence caused one or more extant nodes to change value (from green/superior to red/inferior, or vice versa), the alpha of all nodes immediately adjacent to the changed node was automatically refreshed to 1.0. This effect was carried over to the legend, so that nodes determined to be aged (those with low alpha) were faintly displayed, and thus considered to be “outdated” regimens.

### Historical Representation of Meta-Analytic Network

In order to create the enhancements described above, it was necessary to temporally develop the network, beginning with the first year of publication and proceeding to the most recent year. As a result, visualization of changes in evidence over time was possible.

### General Considerations

The analysis was undertaken using the R statistical programming language (http://www.r-project.org/). iGraph, a freely available package for R and other applications, was used for graph visualization (http://igraph.sourceforge.net/).

## Results

We identified 24 RCTs comparing at least two treatments for newly-diagnosed CML, with *n*=17 substantively identical regimens [8-31]. These are shown chronologically in Table 1. A total of 9700 patients were enrolled across all trials.

**Figure 1:**
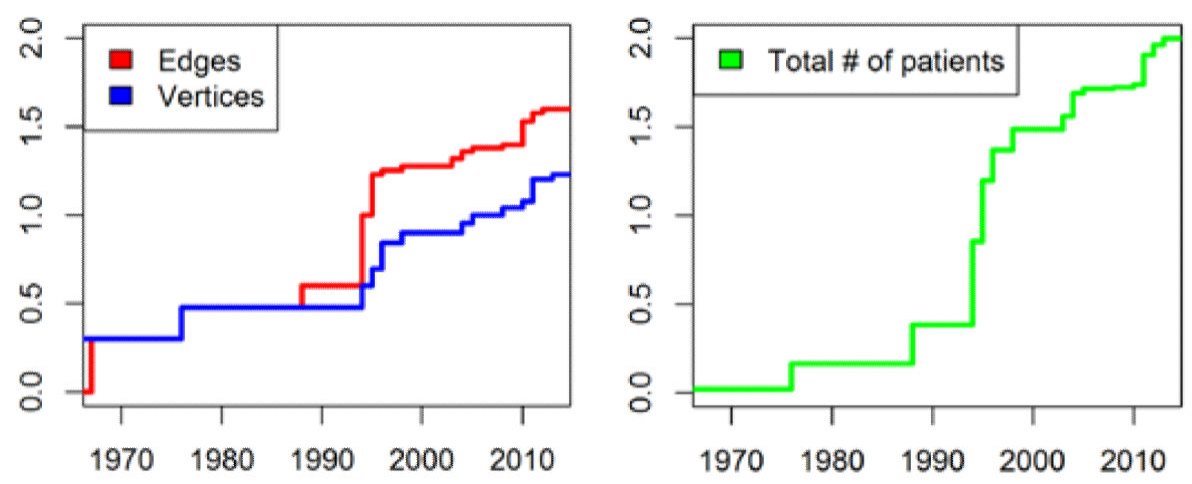
Graph summary statistics, over time. Vertical axes are logarithmic for both panels. In the right panel, total number of patients is normalized to 100 (year 2012)

Imatinib and busulfan were the most highly connected treatment regimens, with degree of 13 in both cases. Five treatments (29%) were singly connected to the network. Additional graph measures are shown in Figure 1.

**Figure 2:**
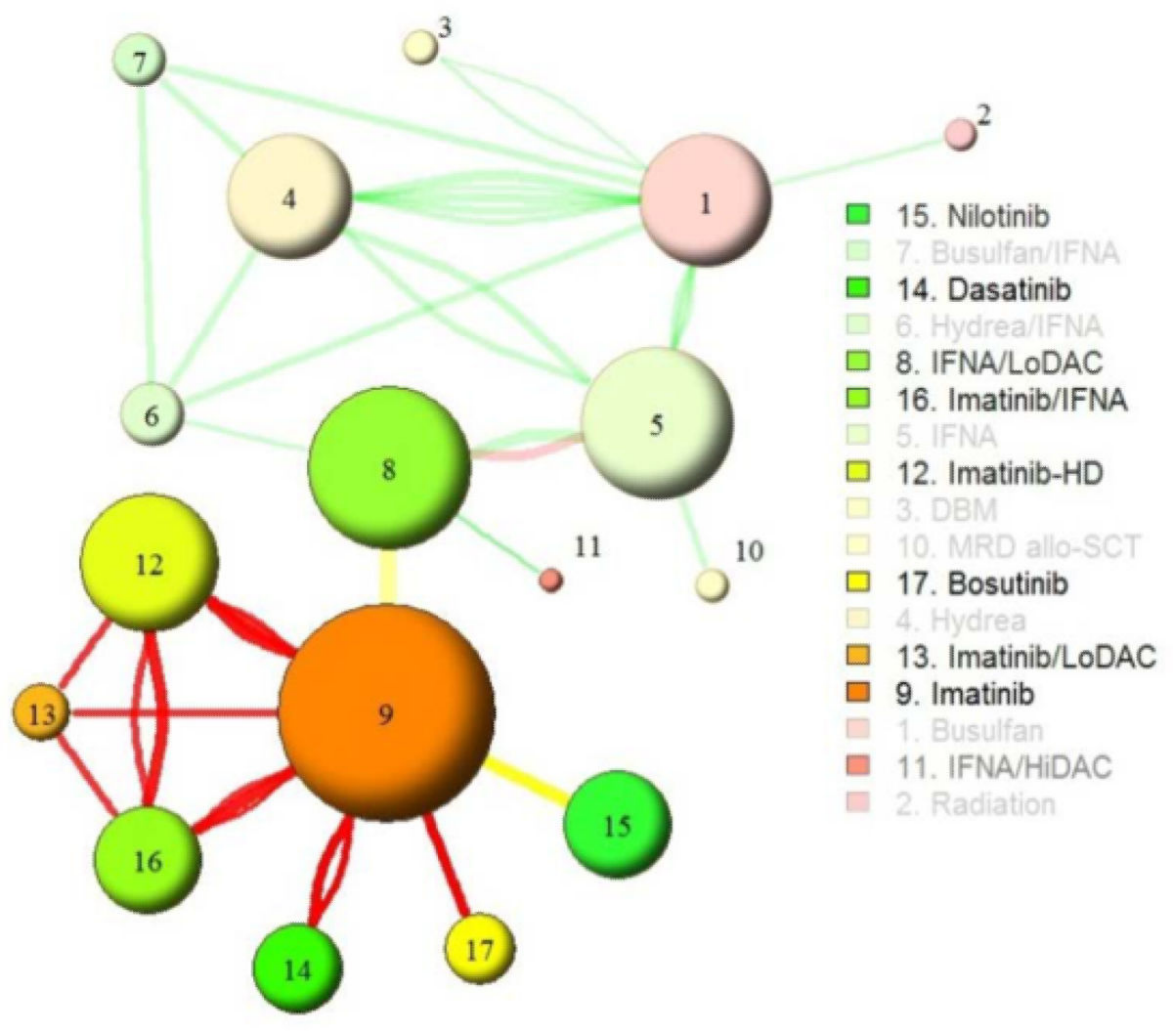
CML primary treatment network analysis, 2012

Figure 2 shows the enhanced graph for the year 2012.

**Figure 3:**
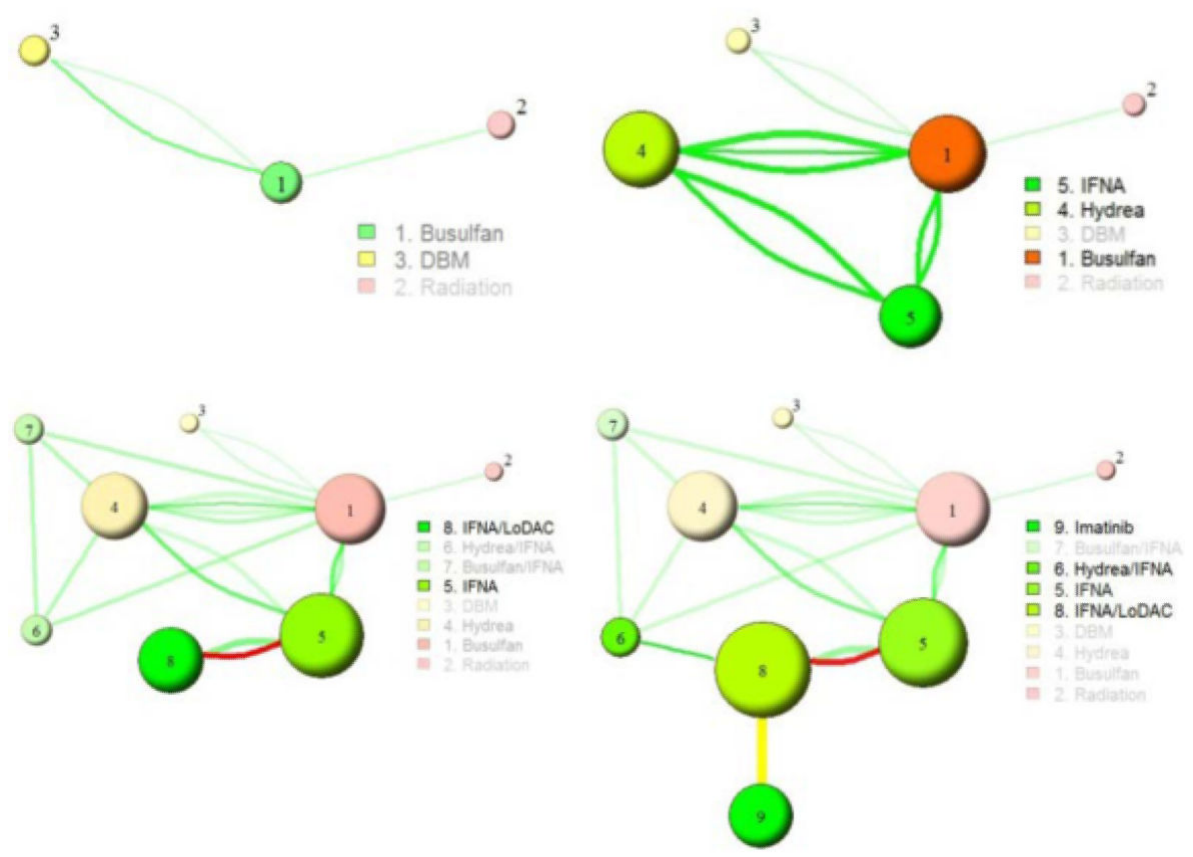
Historic CML primary treatment network analyses for the years 1992, 1994, 2002, 2003

Figure 3 shows four historical representations of the graph. Between 1992 and 1994 (top panels), two new treatments were introduced, and the older “superior” treatment (busulfan) transitioned to an “inferior” status. Between 2002 and 2003 (bottom panels) evidence for imatinib was introduced, and it rose to the top of the “superior” treatment options.

An animated movie of the graph evolution from 1968 to 2012 is freely available at http://hemonc.org/docs/CMLhistory.avi. The R code is freely available upon request.

## Discussion and Conclusion

Several notable conclusions can be made by examining the modern and historical meta-analytic network graphs. First, there is a clear inflection point in the mid-1990’s, after which the number of regimens, clinical trials, and clinical trial participants increased rapidly (Figure 1). Second, overall survival was substituted by surrogate outcomes from 2003 onwards, reflecting the radical improvement in prognosis of CML. While this is welcome news, the general decrease in the quality of the outcome evidence makes interpretation of the modern RCTs more difficult [32]. Third, several distinct “paradigm shifts” can be discerned, based upon the phenomenon of over-turning of previously superior treatment regimens: busulfan in 1994, hydroxyurea in 1995, and imatinib in 2010.

In terms of concordance with the most recent NCCN Guidelines®, our two most superior (and current) regimens, nilotinib and dasatinib, are recommended; the guidelines also suggest consideration of interferon-α for patients intolerant of tyrosine kinase inhibitors (TKI’s) [6]. Imatinib, which is ranked as an inferior regimen in Figure 2, continues to be recommended by the NCCN. Notably, this recommendation hinges primarily on the assertion that imatinib has shown a definitive long-term survival advantage, which is based on historical comparisons, not RCTs (the seminal IRIS trial of imatinib vs. interferon-α/low-dose cytarabine experienced a crossover rate of 90%, making long-term comparisons unreliable) [33, 34]. Because our analysis only includes RCTs, this information is not present in the visualization. This decision to include only high levels of evidence was intentional, although future work will focus on methods of inclusion of historical and contemporaneous comparative effectiveness data.

The treatment for chronic-phase CML has evolved through several eras, which are captured effectively by the modern and historical graphs. In the first era, conventional chemotherapy was the only option; several trials in the early 1990’s established the superiority of hydroxyurea to the standard treatment since the 1940’s, busulfan. From the mid-1990’s, improved mortality was observed with the introduction of interferon-α, ushering in the so-called “interferon era.” The IRIS trial in 2003 led to the “imatinib era,” as shown in Figure 3, lower right panel [19]. Most recently, a series of 2^nd^ and 3^rd^ generation TKI’s, as well as combinations of imatinib with other drugs, have begun to usher in the “post-imatinib era” [35]. Of note, a curative treatment has been available through most of these eras: allogeneic stem cell transplant [36]. As Figure 2 demonstrates, this treatment has rarely been compared against others in a randomized fashion.

There are several important limitations to the current approach. As with any meta-analysis, the results should be interpreted cautiously, since the study populations may differ significantly and publication bias may be present. Additionally, we simplified the valuing of vertices considerably by introducing a win/lose/tie schema, which does not measure the magnitude of outcomes. Future work will explore direct incorporation of outcome magnitudes into the model. We also did not adjust vertex value by indirect comparisons but rather elected to let aged nodes “outdate” through a fading process, with the implications that regimens that have not been studied for some time are unlikely to be a part of current practice. There are clearly exceptions to this rule, such as a regimen whose utility was proven beyond a doubt many years ago. Future work will investigate ways of resolution of these exceptions, as well as application of inheritance rules to the graph. Multiple regimens can contain the same drug(s) and conveyance of this information will require further refinement. Finally, this visualization includes neither comparative effectiveness data nor the other two components of quality measurement: toxicity and cost. In order to make fully informed decisions about optimal treatment strategy, this information is usually taken under consideration; its inclusion in the automated network analysis will also be the focus of future work.

In conclusion, we have demonstrated a new approach to the analysis and visualization of complex clinical data, which does not rely on subjective human interpretation. In the example of primary treatment of CML, the constructed hierarchy closely parallels that developed by human expert consensus. Our method is generalizable and should therefore work with more complicated disease phenotypes and contexts, such as the adjuvant treatment of breast cancer. Once more broadly validated, this automated method has the potential to augment or replace the current approach to guideline development.

## Figures and Tables

**Table 1:** Summary of RCTs

Author (year)	Regimen 1	Regimen 2	
Witts et al. (1968)	Busulfan	Radiation	
Canellos et al. (1975)	Busulfan	DBM	
Silver et al. (1987)	Busulfan	DBM	
Hehlmann et al. (1993)	Busulfan	Hydrea	
Tura et al. (1994)	Busulfan	Hydrea	IFNA
Hehlmann et al. (1994)	Busulfan	Hydrea	IFNA
Allan et al. (1995)	Busulfan	Hydrea	
	Busulfan/IFNA	Hydrea/IFNA	
Ohnishi et al. (1995)	Busulfan	IFNA	
Guilhot et al. (1997)	IFNA	IFNA/LoDAC	
Baccarani et al. (2002)	IFNA	IFNA/LoDAC	
Kuhr et al. (2003)	Hydrea/IFNA	IFNA/LoDAC	
O’Brien et al. (2003)	IFNA/LoDAC	Imatinib	
Ohnishi et al. (2004)	IFNA	MRD allo-SCT	
Olsson et al. (2004)	Busulfan	Hydrea	
Deenik et al. (2007)	IFNA/HiDAC	IFNA/LoDAC	
Baccarani et al. (2009)	Imatinib	Imatinib-HD	
Cortes et al. (2010)	Imatinib	Imatinib-HD	
Kantarjian et al. (2010)	Dasatinib	Imatinib	
Preudhomme et al. (2010)	Imatinib	Imatinib-HD	
	Imatinib/IFNA	Imatinib/LoDAC	
Saglio et al. (2010)	Imatinib	Nilotinib	
Hehlmann et al. (2011)	Imatinib	Imatinib- HD	Imatinib/ IFNA
Simonsson et al. (2011)	IFNA/Imatinib	Imatinib	
Cortes et al. (2012)	Bosutinib	Imatinib	
Radich et al. (2012)	Dasatinib	Imatinib	

DBM: dibromomannitol; Hydrea: hydroxyurea; IFNA: interferon-α; Lo/HiDAC: low-/high-dose cytarabine; MRD allo-SCT: matched related donor allogeneic stem cell transplant; HD: high dose
